# Partial coupling delay induced multiple spatiotemporal orders in a modular neuronal network

**DOI:** 10.1371/journal.pone.0177918

**Published:** 2017-06-01

**Authors:** XiaoLi Yang, HuiDan Li, ZhongKui Sun

**Affiliations:** 1 College of Mathematics and Information Science, Shaanxi Normal University, Xi’an, PR China; 2 Department of Applied Mathematics, Northwestern Polytechnical University, Xi’an, PR China; Lanzhou University of Technology, CHINA

## Abstract

The influence of partial coupling delay on the spatiotemporal spiking dynamics is explored in a modular neuronal network. The modular neuronal network is composed of two subnetworks which present the small-world property and scale-free property, respectively. Numerical results show that spatiotemporal order that the modular network is most coherent in time and nearly synchronized in space can emerge intermittently when the coupling delays among neurons are appropriately tuned. The appropriately tuned delays are further detected to be integer multiples of the intrinsic spiking period of the modular neuronal network, which implies that the phenomenon of multiple spatiotemporal orders could be the result of a locking between the length of coupling delay and the intrinsic spiking period of the modular neuronal network. Moreover, the multiple spatiotemporal orders are verified to be robust against variations of the fraction of delayed connection as well as the key parameters of network architecture such as the rewiring probability, the average degree of small-world subnetwork, the initial nodes of scale-free subnetwork and the total size of the modular network.

## Introduction

Neurons are the basic function units of neuronal network. In recent years, there has been a growing interest in neurodynamics on neuronal network [1, 2]. Previous studies have shown that several factors, including noise, coupling delay and connection configuration, can shape the neuronal firing pattern significantly. As for noise, it is well known that neurons are inevitably affected by all kinds of noise, which arises from many different sources, such as quasi-random release of neurotransmitter by the synapses, a random switching of ion channels and random synaptic input from other neurons [3, 4]. A series of examples have demonstrated that noisy fluctuations can interact with the neuron’s nonlinearities to render some counterintuitive effects. To be mentioned here are synchronization induced by external noise, stochastic resonance, coherence resonance and noise-induced order. Especially interesting is the phenomenon of coherence resonance [5] that a nonlinear excitable system spikes regularly when driven by purely noise. This has been confirmed by numerous numerical simulations in various neuronal networks [6–13], ranging from regular neuronal network, small-world neuronal network and scale-free neuronal network to spatially extended neuronal system.

Besides noise, coupling delay is another important factor affecting the dynamics of neural systems. The diverse roles of coupling delay on the firing dynamics have been widely investigated. Wang et.al. have reported appropriately tuned delays can induce multiple coherence resonances in inhibitory and excitatory coupled Hodgkin-Huxley (HH) neurons [14]. Multiple stochastic resonances in an intermittent fashion induced by coupling delays have been revealed in small-world and scale-free neuronal network, respectively [15–17]. In regular network of Hindmarsh-Rose (HR) neurons, intermediate coupling delay has been found to make ordered spatial patterns emerge [18]. The dramatic influences of coupling delay attached to chemical or electrical synaptic connections on synchronous property of neuronal firing have been revealed recently. For example, synchronization in neuronal network with coupling delay can occur at lower coupling strength compared to that with instantaneous coupling [19], which implies that synchronizability of coupled neurons can be enhanced by coupling delay. In Newman-Watts (NW) network of HH neurons [20], it has been found that coupling delay can induce synchronization transitions when the channel noise intensity is intermediate. Delay was also revealed to induce a transition from zigzag fronts to clustering anti-phase synchronization and further to regular in-phase synchronization in small-world network of the Rulkov maps [21]. Furthermore, temporal coherence together with spatial synchronization has found to be simultaneously enhanced by an appropriate coupling delay in noisy neuronal networks [22, 23]. In addition, noises and coupling delay also always coexist in many natural systems such as ecosystems, biological systems and physical systems. The combinations of noise and delay often change fundamentally dynamics of the systems. Interested readers may refer to Refs. [24–31].

In addition, the connection configuration of consistent neurons in neuronal network is also a contributing element for shaping neuronal dynamics. Statistical analysis and experimental evidence has implied that the connection structure in the cerebral cortex had small-world characteristics. The computational model of neuronal network with small-world property has been predominant in the literature, characterized by a small value of normalized path length and a comparatively large value of clustering coefficient. On the other hand, recent neuroanatomic studies have revealed that neurons with similar functional and connectional features are arranged into distinctive modules [32, 33]. The modules are areas in which neurons are densely connected to other neurons within the same module but sparsely connected to neurons in other modules. Now there has been a growing interest in the firing dynamics of such modular neuronal network [34–39]. For instance, diversity-induced stochastic resonance has been reported in a modular neuronal network consisting of several small-world subnetworks, and this kind of stochastic resonance can appear intermittently with the increase of coupling delay [34]. The bursting dynamics of neurons in a modular network have been discussed, in which a critical coupling strength to obtain burst synchronization in terms of intra- and inter- connection probability has been presented [35]. The phenomenon of delay-induced synchronization was found to occur in a modular network with hybrid synapses, where the synchronization of neuron activity can be either promoted or destroyed as the coupling delay increases [36]. Ref. [37] has investigated suppression of bursting synchronization by differential feedback control and analyzed the nontrivial influence of coupling delay on differential feedback control of bursting synchronization in a modular neuronal network.

Note that a characteristic feature for the above mentioned delay is that coupling delay is introduced to every connection of coupled neurons. For a neuronal system in reality, just as what has been indicated in Ref. [40], propagation speed of neuronal information is mostly dependent on the length, the diameter, and the kind of the axons between the neurons. It may thus be inferred that information communications between some neurons are instantaneous or with some negligible delay and the others are not instantaneous. The case that only part of neuronal connections are delayed is termed as partial coupling delay for convenience. Effect of partial coupling delay on resonant dynamics has been discussed in a small-world network of Rulkov neurons, where stochastic multi-resonance is observed as coupling delay is increased [40]. Apart from this, there is hardly any other report concerning the interplay of partial coupling delays and firing dynamics for coupled neurons, and this issue is still puzzled now.

Inspired by the above findings, the present study therefore focuses on how the partial coupling delay, influences the spatiotemporal dynamics of temporal coherence and spatial synchronization in a modular neuronal network. To the best of our knowledge, there has been no corresponding work contributing to this subject until recently. The remainder of this study is structured as follows. Firstly, the mathematical model of the concerned modular network and measurement for spatiotemporal order are presented, respectively. Then the main result about multiple spatiotemporal orders are illustrated. Finally, we present a conclusion of this study.

## Mathematical model and measurement

### Mathematical model

To begin with, a modular network is constructed in the following way. Firstly, one generates *M* subnetworks each of which could be regular, small-world or scale-free network. Then, nodes in different subnetworks are randomly connected with a given inter-module probability *p*_*out*. By doing this, a framework of modular network is constructed. If each subnetwork contains equal number of nodes *N*_0_, the total size of the modular network is thus *N* = *MN*_0_. The two-dimensional Rulkov map [41] is employed to simulate the local dynamics of each neuron, then the discrete equations for the modular network are defined as:

$$\{\begin{array}{ll} x_{I,i}(n+1) & =\frac{\alpha }{1+x_{I,i}^{2}(n)}+y_{I,i}(n)+g\_in\sum_{j}A_{I}(i,j)(x_{I,j}(n-\tau )-x_{I,i}(n))\\ & +g\_out\sum_{J}\sum_{j}B_{I,J}(i,j)(x_{J,j}(n-\tau )-x_{I,i}(n))+D\xi_{I,i}(n)\\ y_{I,i}(n+1) & =y_{I,i}(n)-\beta x_{I,i}(n)-\sigma \end{array}$$ (1)

in which the subscript pair (*I*,*i*) represents the *ith*(*i* = 1,2,…,*n*) neuron in the *Ith*(1,2,…,*M*) subnetwork, and *n* is the discrete-time index. *x*_*I*,*i*_ and *y*_*I*,*i*_ are fast and slow dynamical variables of the Rulkov map, which denote the membrane potential and the variation of ion concentration, respectively. *α* is the main control parameter of Rulkov map, and the isolated neuron can exhibit different firing behaviors if appropriately adjusting its value [41]. If *α*<2.0, the neuron has a stable fixed point. (x* = −1,y* = −(2+*α*)/2). In this case the neuron is excitable for it can produce spiking pulse by external stimuli. If *α*>2.0, bursting oscillation emerges via a Hopf bifurcation. Here, we set *α* = 1.99 to guarantee each neuron is excitable. *β* and *σ* are small parameters. ξ_*I*,*i*_(*n*) denotes independent Gaussian noise, whose statistical property obeys 〈ξ_*I*,*i*_(*n*)〉 = 0,〈ξ_*I*,*i*_(*n*)ξ_*J*,*j*_(*n*′)〉 = *δ*_*IJ*_*δ*_*ij*_*δ*(*n−n*′).*D* is the noise intensity. *τ* denotes coupling delay, and *p*_*delay* indicates the fraction of delayed connection. That is to say, the fraction of delayed connection is determined by a probability *p*_*delay* and the coupling delay *τ* between connected neurons is not equal to zero with probability *p*_*delay*. In detail, one extreme case of *p*_*delay = 0* implies that information communications between all connected neurons is instantaneous, i.e., *τ = 0* is for connected neurons. The other extreme case of *p*_*delay = 1*.*0* indicates that information communications between all connected neurons is not negligible, i.e., *τ*≠0 is for all connected neurons. The intermediate case of 0<*p*_*delay<1* implies that only part of neuronal connections are delayed, i.e, the portion of delayed connection is *p*_*delay*. *g*_*in* and *g*_*out* depict intra-coupling strength and inter-coupling strength, respectively. *A*_*I*_ = (*A*_*I*_(*i*,*j*)) is an intra-connection matrix for the *Ith* subnetwork: *A*_*I*_(*i*,*j*) = *A*_*I*_(*j*,*i*) = 1 if neuron *i* is connected to neuron *j* inside the *Ith* subnetwork, otherwise *A*_*I*_(*i*,*j*) = *A*_*I*_(*j*,*i*) = 0, and *A*_*I*_(*i*,*i*) = 0. *B*_*I*_ = (*B*_*I*_,_*J*_(*i*,*j*)) is a inter-connection matrix: *B*_*I*_,_*J*_(*i*,*j*) = *B*_*J*,*I*_(*j*,*i*) = 1 if the *ith* neuron in the *ith* module is connected to the *jth* neuron in the *Jth* module, otherwise *B*_*I*_,_*J*_(*i*,*j*) = B_*J*,*I*_(*j*,*i*) = 0.

In this study, the considered modular network consists of two subnetworks, whose connection architecture follows the procedure of scale-free network [42] and small-world network [43], respectively. For the small-world subnetwork, starting from a regular ring of *N*_0_ nodes with periodic boundary condition, each node is connected to its *k* nearest neighbors (*k* is referred to be the average degree), and then every edge is rewired with rewiring probability *p*_*in*. For the scale-free subnetwork, starting with a small number *m*_0_ of fully connected nodes, at every time step a new node is added, and then link this new node to *m* different nodes that already exists. Repeating this procedure *N*_0_-*m*_0_ times until a scale-free network with *N*_0_ nodes is generated. Unless otherwise stated, the parameters of the modular network is set as: *β* = *σ* = 0.001, *g*_*in* = *g*_*out* = 0.005, *p*_*out* = 0.05, *N*_0_ = 80, *M* = 2, *D* = 0.0018, *m* = *m*_0_ = 3, *p*_*in* = 0.1, *k* = 6. Due to the randomness of this modular network, the following result is averaged over 20 different realizations to ensure the reliability of numerical results.

### Measurement

In order to quantitatively characterize the spatiotemporal dynamics of temporal coherence and spatial synchronization in the modular neuronal network, we introduce the coefficient of variation *R* and the standard deviation *σ*, respectively. *R* is defined as follows [13]:

$$R=\sum_{I,i}R_{I,i}\text{ with }R_{I,i}=\frac{\sqrt{Var(T_{k}^{I,i})}}{{\langle T_{k}^{I,i}\rangle }_{}}$$ (2)

where $T_{k}^{I,i}=t_{k+1}^{I,i}-t_{k}^{I,i}$ represents the interspike intervals (ISIs) of the *ith* neuron in the *Ith*(1,2,…,*M*) subnetwork, $t_{k}^{I,i}$ is the time of the *kth* spike for the *ith* neuron in the *Ith*(1,2,…,*M*) subnetwork, 〈⋅〉denotes average over time. Obviously, the coefficient of variation *R* can measure temporal coherence of the modular network quantitatively, and smaller *R* implies higher regularity of the spike train. The standard deviation *σ* is introduced as follows [23]:

$$\sigma =\langle \sigma \left(n\right)\rangle \text{ with }\sigma (n)=\sqrt{\frac{\left(\underset{I,i}{\sum }x_{I,i}^{2}\left(n\right)\right)/N-{\left(\underset{I,i}{\sum }x_{I,i}\left(n\right)/N\right)}^{2}}{N-1}}$$ (3)

Clearly, smaller *σ* indicates higher spatial synchronization of the modular network.

If the spatiotemporal behavior of the modular network is most coherent in time and nearly synchronized in space, the modular network is claimed to be spatiotemporal order. That is to say, spatiotemporal order will emerge when *R* and *σ* simultaneously obtain their minimums.

## Multiple spatiotemporal orders induced by partial coupling delay

This section investigates how the partial coupling delay influences the spatiotemporal dynamics of temporal coherence and spatial synchronization in the above mentioned modular neuronal network. The parameters of coupling delay *τ* and the fraction of delayed connection *p*_*delay* are taken as control variable.

**Fig 1:**
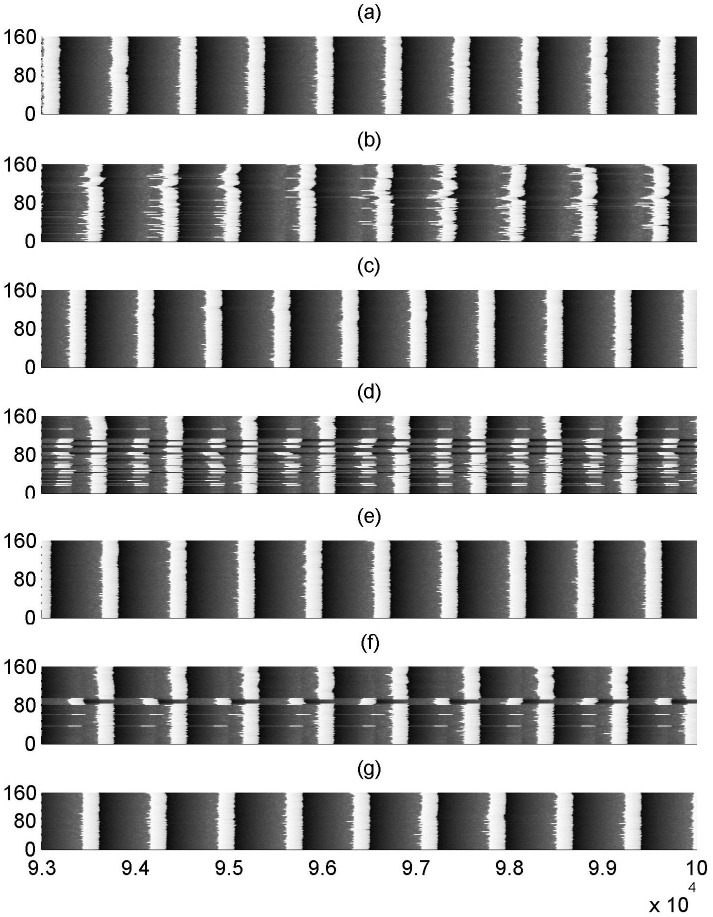
Spatiotemporal plots of the modular neuronal network for different coupling delays when *p*_*delay* = 0.1. (a)*τ* = 0, (b)*τ* = 500, (c)*τ* = 720, (d)*τ* = 1260, (e)*τ* = 1440, (f)*τ* = 2000, (g)*τ* = 2160.

Firstly, we discuss how the spiking behavior evolves with coupling delay *τ*. Fig 1 illustrates some typical spatiotemporal plots for different *τ* when the fraction of delayed connection is fixed at *p*_*delay* = 0.1. When the coupling delay is absent (i.e., *τ* = 0), as showed in Fig 1(a), the spikes for all the neurons are most coherent in time and nearly synchronized in space, which indicates the spatiotemporal order can occur in the modular neuronal network. As *τ* is increased to *τ* = 500 (please see Fig 1(b)), some spikes become apparently irregular and non-synchronous, which implies that spatiotemporal order is destroyed by the presence of coupling delay. Interestingly, as *τ* is further increased to *τ* = 720, the performance of spatiotemporal order reappears (please see Fig 1(c)). Hereafter, the phenomenon of spatiotemporal order alternately appears at *τ* = 1260,1440,2000,2160 (please see Fig 1(d)–1(g)). The evolution of spatiotemporal plots obviously shows that spatiotemporal order can intermittently occur with varying coupling delays. That is to say, multiple spatiotemporal orders can be induced by partial coupling delay in the modular neuronal network.

**Fig 2:**
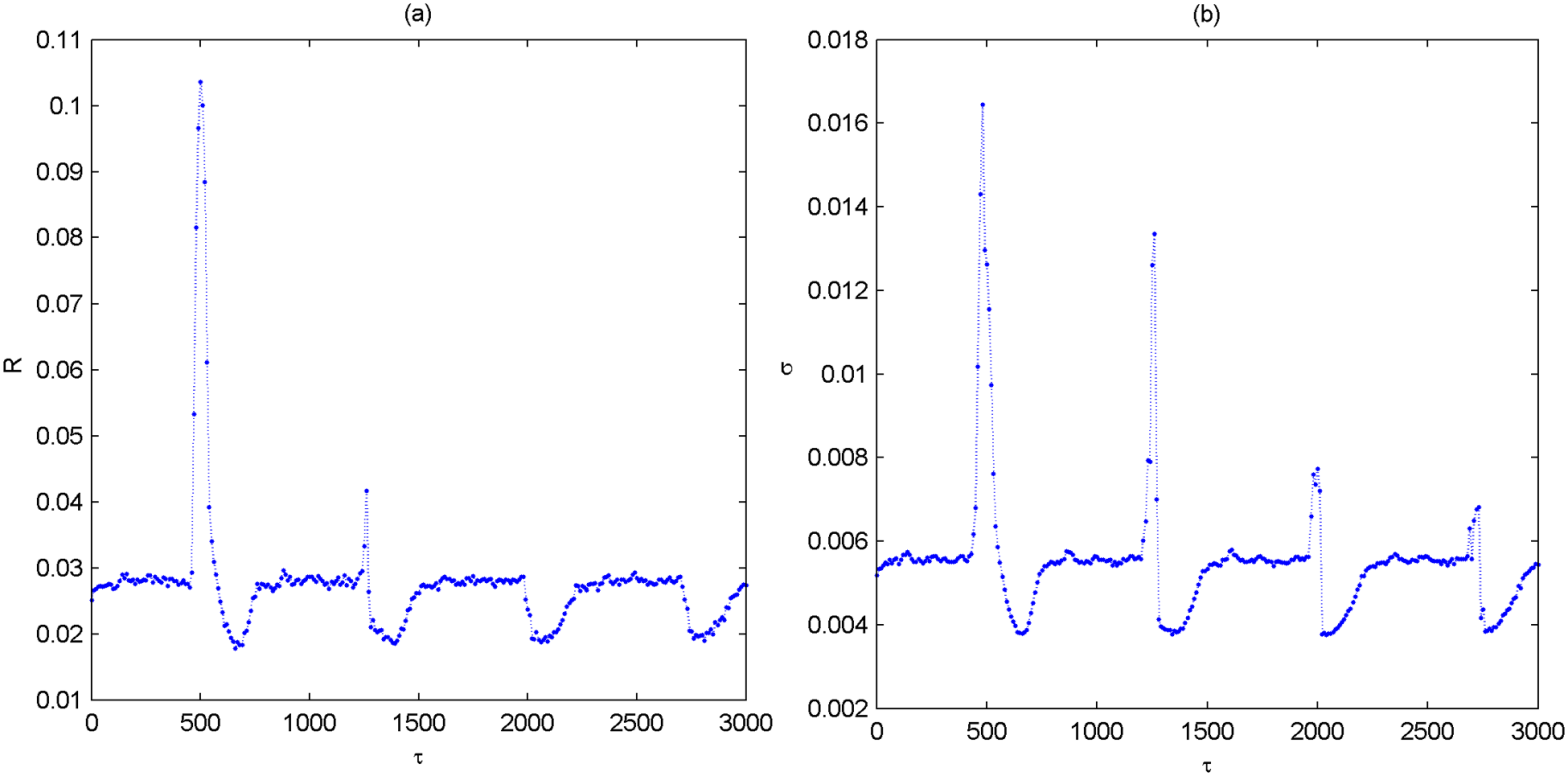
Dependence of the coefficient of variation *R* and the standard deviation *σ* on coupling delay *τ* when *p*_*delay* = 0.1. (a) Dependence of *R* on *τ*. (b) Dependence of σ on *τ*.

To characterize how the partial coupling delay affects the spiking behavior quantitatively, Fig 2 depicts the evolution of the coefficient of variation *R* and the standard deviation *σ* with varying coupling delay *τ*, respectively. From Fig 2(a), one can see that *R* passes through several minimums with the increase of *τ*, which means that the optimal regularity of the spikes in the modular neuronal network occurs intermittently. At the same time, the evolution of *σ*, displayed in Fig 2(b), also undergoes several minimums with the increase of *τ*, which indicates the optimal synchronization of the spikes in the modular neuronal network also occurs intermittently. Surprisingly, the tendency of curve *σ*~*τ* is similar with that of curve *R*~*τ*, moreover, the minimums of *R* and *σ* almost appear at the same values of delay (that is about τ = 0,720,1440,2160). Combined with these two figures, one can conclude that spatiotemporal order, i.e., the neurons are most coherent in time and nearly synchronized in space, can intermittently appear when the coupling delays are appropriately tuned. This characteristic is consistent with what is stated in Fig 1.

**Fig 3:**
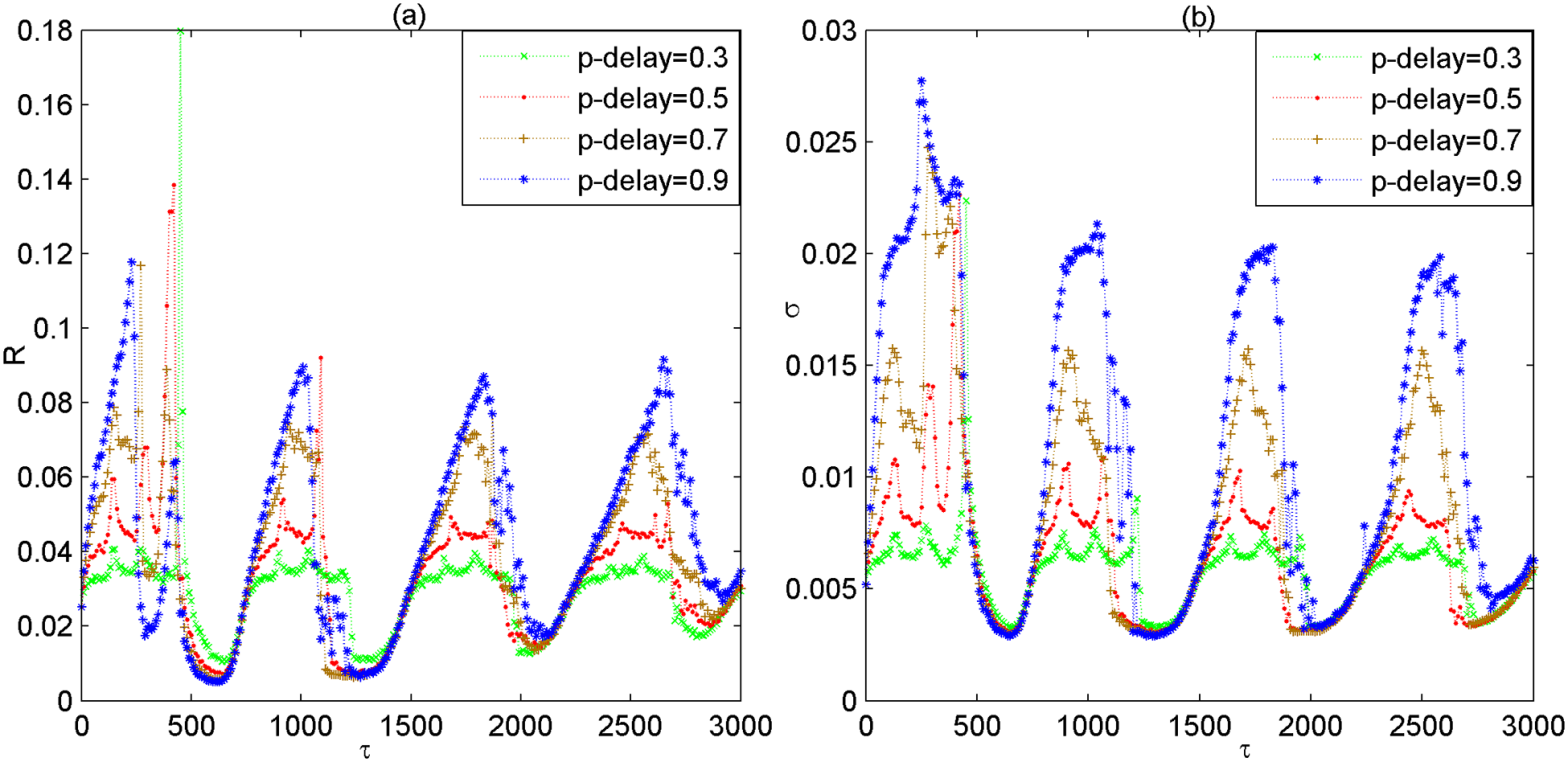
Dependence of the coefficient of variation *R* and standard deviation *σ* on coupling delay *τ* for different values of *p*_*delay*. (a) Dependence of *R* on *τ*. (b) Dependence of *σ* on *τ*. Here, *p*_*delay* = 0.3,0.5,0.7,0.9.

Meanwhile, when the fraction of delayed connection is changed to other values (e.g., *p*_*delay* = 0.3,0.5,0.7,0.9), the dependence of *R* and *σ* on *τ* is also depicted in Fig 3, respectively. Through a careful inspection of Fig 3, one can see that both the *R*~*τ* curve and the *σ*~*τ* curve simultaneously undergo several minimums in the course of *τ*. Thus, one can declare that the phenomenon of partial coupling delay induced multiple spatiotemporal orders is robust to changes of the fraction of delayed connection.

**Fig 4:**
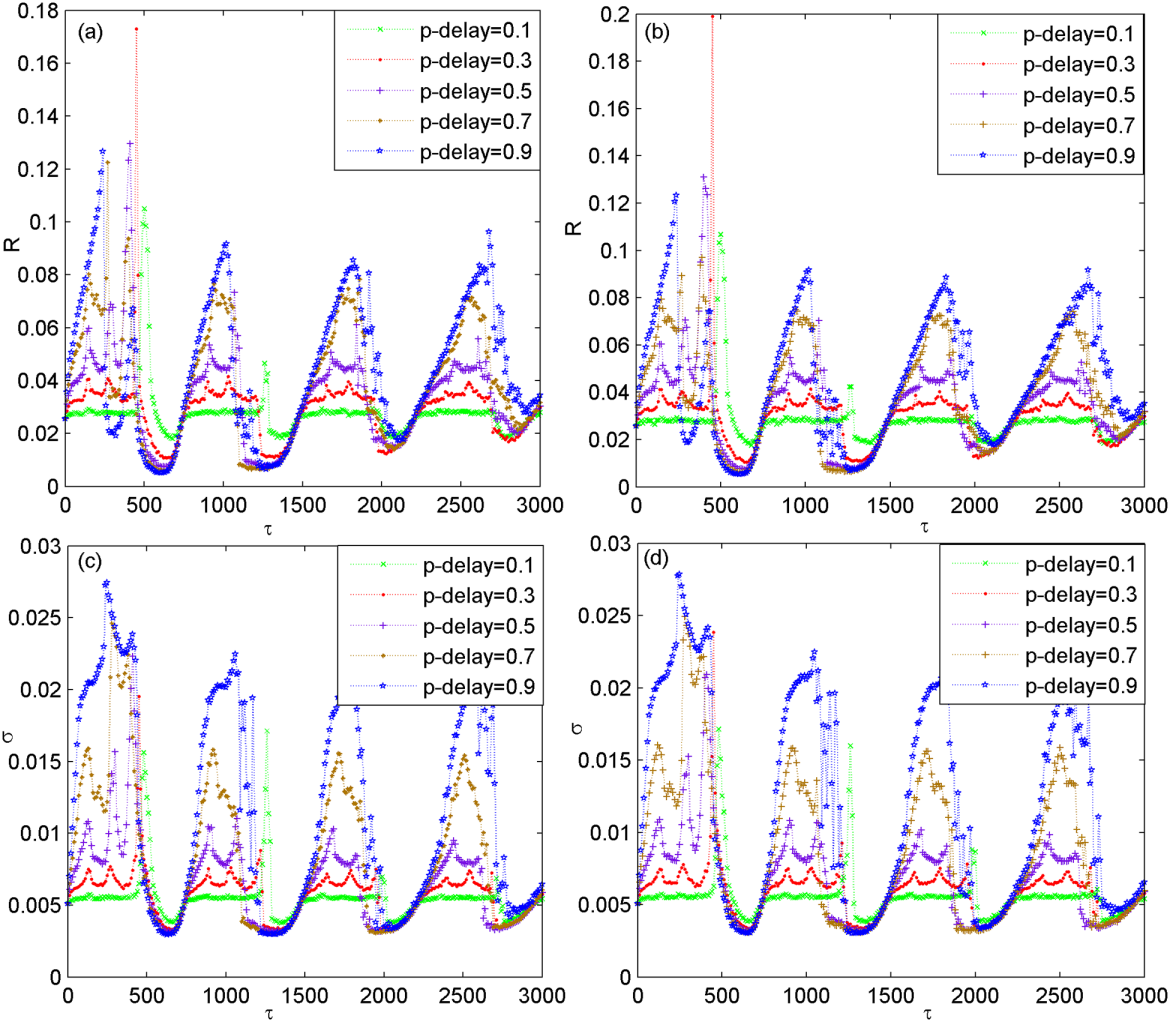
Dependence of the coefficient of variation *R* and the standard deviation *σ* on coupling delay *τ* for different *p*_*delay* and different *p*_*in*. (a) and (c) are for *p*_*in* = 0.3. (b) and (d) are for *p*_*in* = 0.7. Here, *p*_*delay* = 0.1,0.3,0.5,0.7,0.9.

**Fig 5:**
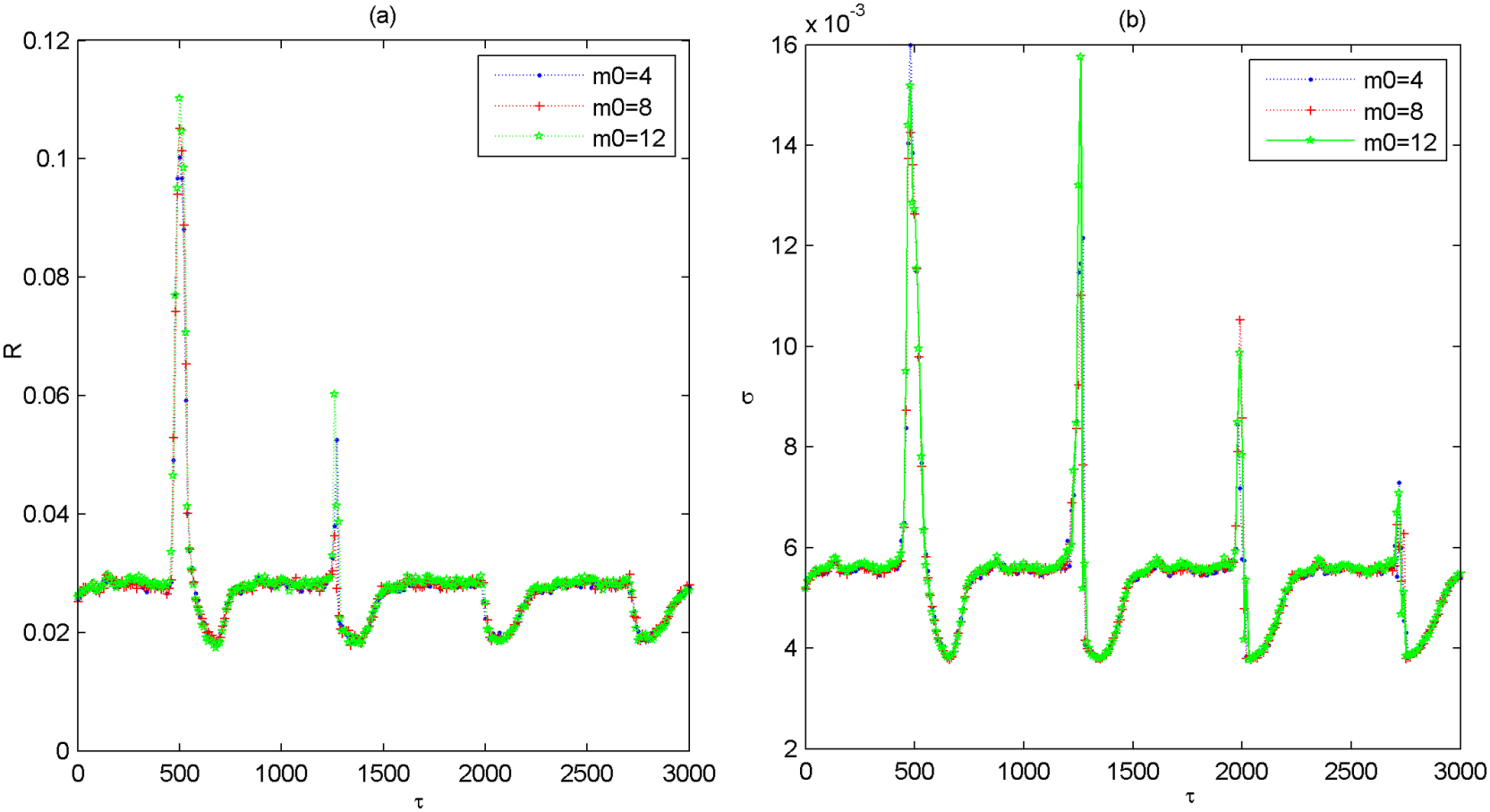
Dependence of the coefficient of variation *R* and the standard deviation *σ* on coupling delay *τ* for different values of *m*_0_ when *p*_*delay* = 0.1. (a) Dependence of *R* on *τ*. (b) Dependence of *σ* on *τ*. Here, *m*_0_ = 4,8,12.

**Fig 6:**
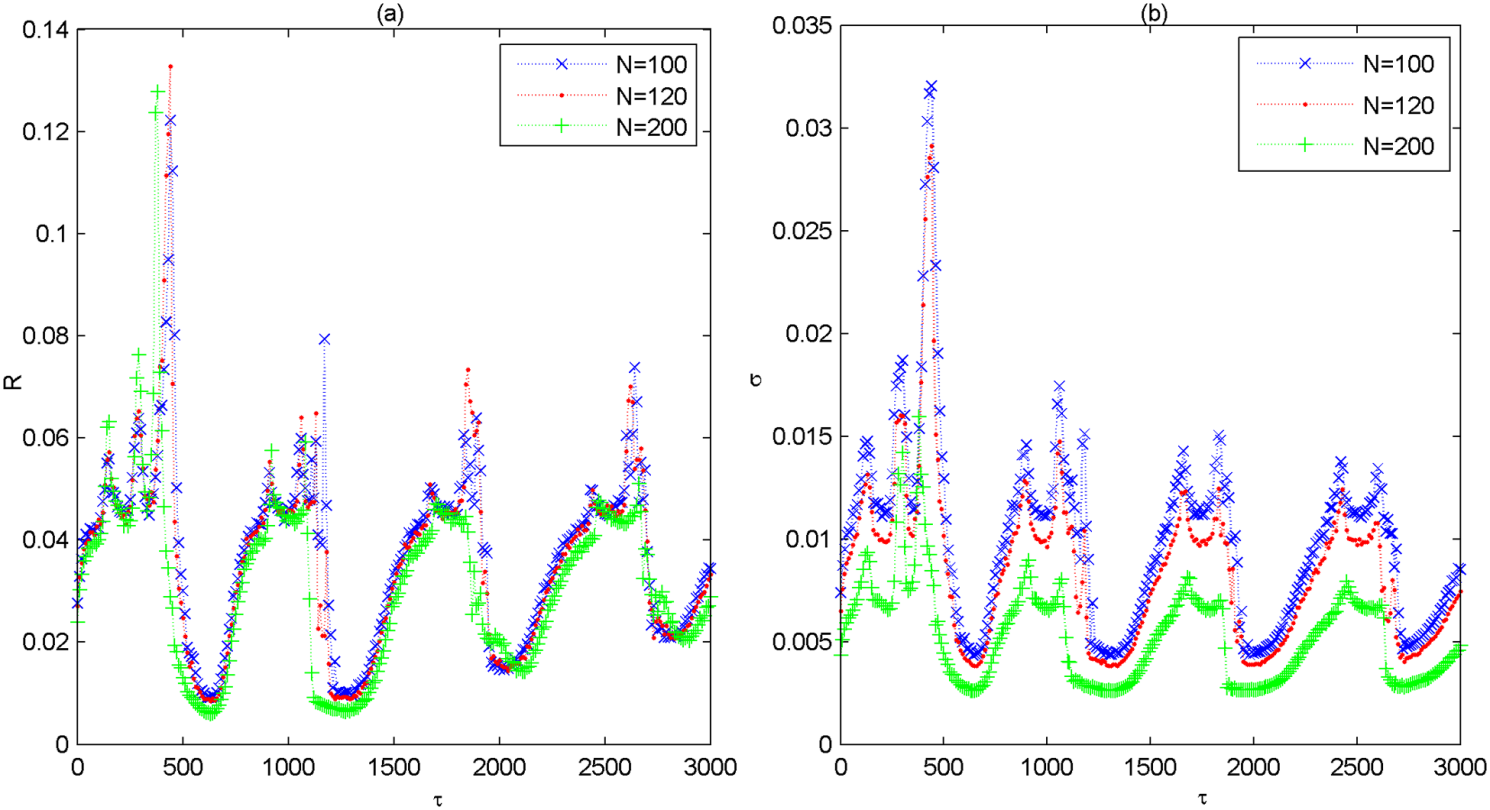
Dependence of the coefficient of variation *R* and the standard deviation *σ* on coupling delay *τ* for different values of *N* when *p*_*delay* = 0.5. (a) Dependence of *R* on *τ*. (b) Dependence of *σ* on *τ*. Here, *N* = 100,120,200.

When the network architecture is changed, e.g., the rewiring probability of small-world subnetwork is changed to other values such as *p*_*in* = 0.3,0.7, the variations of *R* and *σ* as a function of *τ* for different *p*_*delay* is further presented in Fig 4. Left panels are for the case of *p*_*in* = 0.3 and right panels are for the case of *p*_*in* = 0.7, respectively. One can find that the characteristic feature that both *R* and *σ* simultaneously pass through several minimums with the increase of *τ* still appears. This indicates that the phenomenon of multiple spatiotemporal orders induced by partial coupling delay in the modular neuronal network is a robust phenomenon, which also occurs largely independent of the rewiring probability of small-world subnetwork. Additionally, we have verified the robustness of partial coupling delay induced multiple spatiotemporal orders when the scale-free subnetwork structure and the total size of the modular network are changed, respectively. Without loss of generality, the variations of *R* and *σ* as a function of *τ* are respectively illustrated in Fig 5(a) and 5(b) when the initial nodes *m*_0_ of scale-free subnetwork is changed to other values of *m*_0_ = 4,8,12. Meanwhile, Fig 6(a) and 6(b) depicts the dependence of *R* and *σ* on *τ* for different values of system size *N* such as *N* = 100,120,200. From these figures, it is found that *R* and *σ* simultaneously pass through several minimums when the partial coupling delay is adjusted to about *τ* = 0,720,1440,2160, which indicates that the phenomenon of multiple spatiotemporal orders is also robust against the scale-free subnetwork structure and the total size of the modular neuronal network.

**Fig 7:**
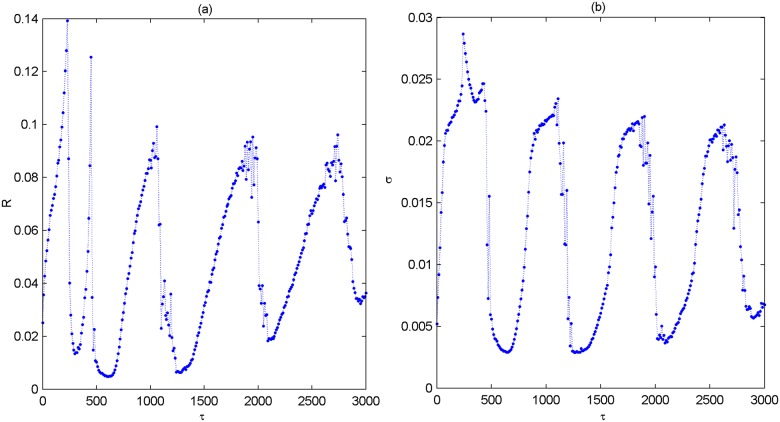
Dependence of the coefficient of variation *R* and the standard deviation *σ* on coupling delay *τ* when *p*_*delay* = 1.0. **(a)** Dependence of *R* on *τ*. (b) Dependence of *σ* on *τ*.

The above results illustrate that the partial coupling delay can induce multiple spatiotemporal orders in the modular neuronal network. We wonder whether this phenomenon can still occur when all connections are delayed, i.e., *p*_*delay* = 1.0. In this case, the coefficient of variation *R* as well as the standard deviation *σ* is firstly employed to quantitatively characterize the spiking behavior of the modular neuronal network. Fig 7 displays the dependence of *R* and *σ* on *τ*, respectively. Fig 7(a) demonstrates that *R* rises and drops repeatedly, intermittently passing through several minimums with the increase of *τ*. Meanwhile, as showed in Fig 7(b), the evolution of *σ*, similar with that of *R*, also rises and drops repeatedly and undergoes several minimums intermittently. More intriguingly, *R* and *σ* simultaneously achieve their minimums at about *τ* = 0,720,1440,2160. The result indeed implies that the phenomenon of multiple spatiotemporal orders still appear when all connections in the modular neuronal network are considered to be delayed.

**Fig 8:**
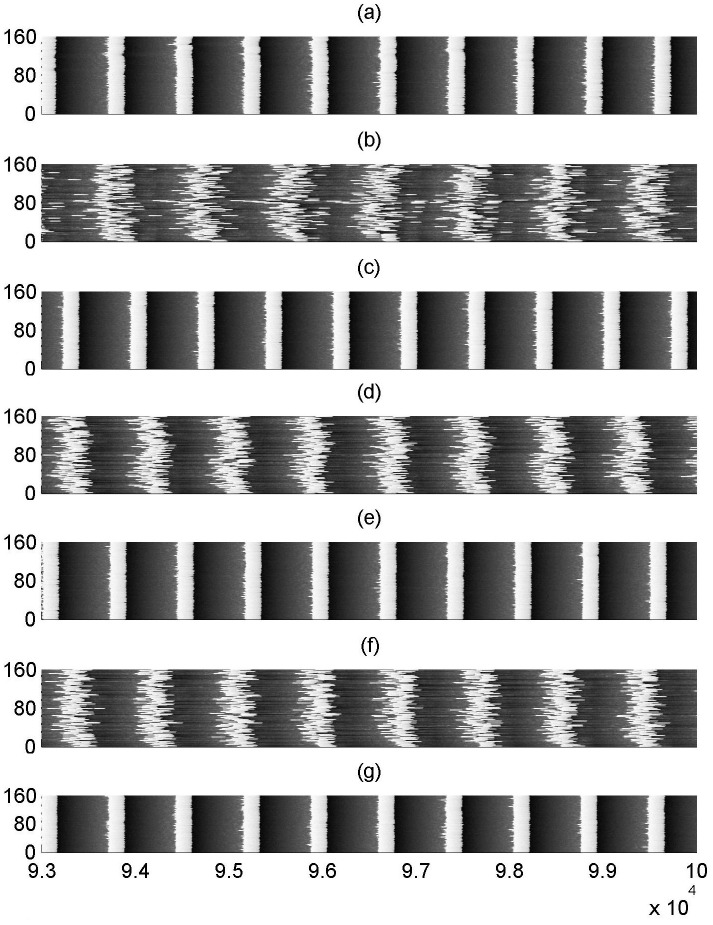
Spatiotemporal plots of the modular neuronal network for different coupling delays when *p*_*delay* = 1.0. (a) τ = 0, (b)τ = 230, (c)τ = 720, (d)τ = 1000, (e)τ = 1440, (f)τ = 1900, (g)τ = 2160.

**Fig 9:**
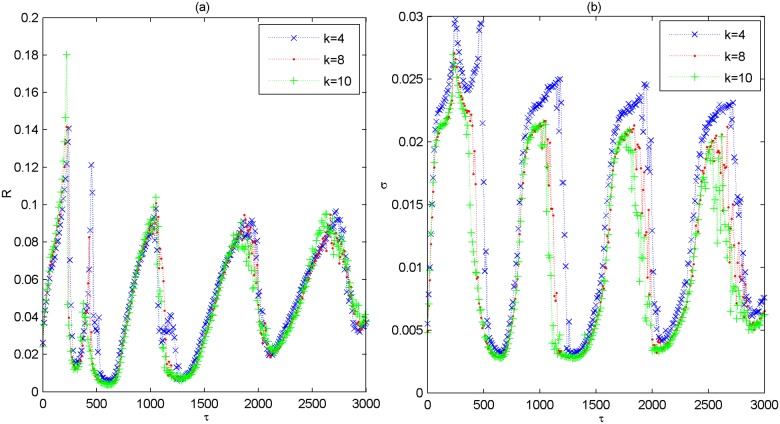
Dependence of the coefficient of variation *R* and the standard deviation *σ* on coupling delay *τ* for different values of *k* when *p*_*delay* = 1.0. (a) Dependence of *R* on τ. (b) Dependence of *σ* on τ. Here, *k* = 4,8,10.

The phenomenon of multiple spatiotemporal orders displayed in Fig 7 is further verified by some typical spatiotemporal plots. From Fig 8, it is seen that the spikes of the modular network is most coherent in time and nearly synchronized in space when *τ* = 0,720,1440,2160 (please see Fig 8(a), 8(c), 8(e) and 8(g)). This is a characteristic feature of spatiotemporal order. Nevertheless, when *τ* = 230,1000,1900 (please Fig 6(b), 6(d) and 6(f)), the spatiotemporal order apparently can’t appear, and the spikes become quite irregularly and non-synchronous. This accords with the results displayed in Fig 7, which further verifies that multiple spatiotemporal orders can still occur when the connections are all delayed. In the following, we change the average degree *k* of small-world subnetwork to other values of *k* = 4,8,10, and depict the variations of *R* and *σ* as a function of *τ* in Fig 9. Similar with that of *k* = 6, *R* and *σ* simultaneously pass through several minimums intermittently. Thus, one can claim that the phenomenon of multiple spatiotemporal orders in the modular neuronal network is robust against changes of the average degree of small-world subnetwork.

**Fig 10:**
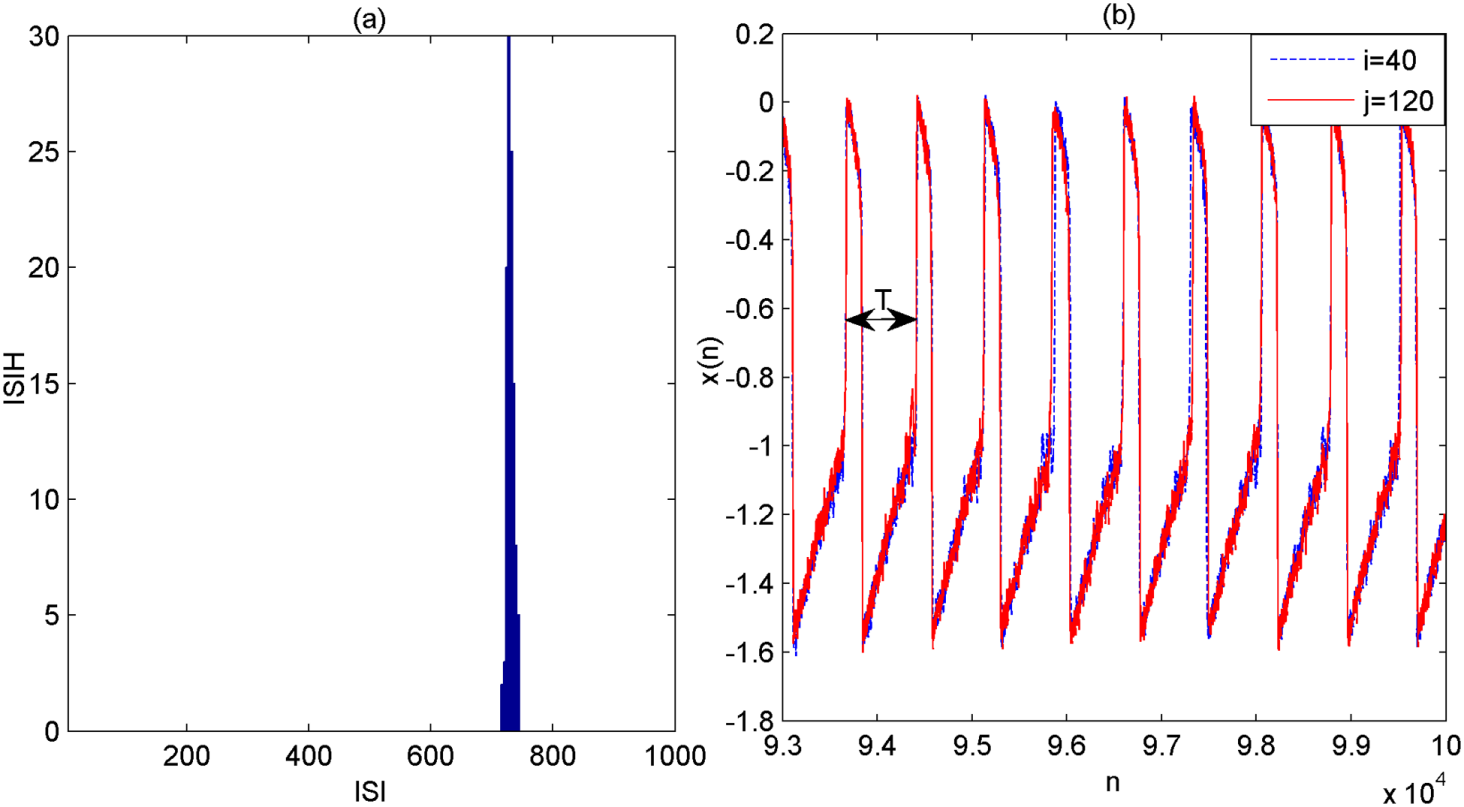
The firing dynamics of the modular neuronal network in the absence of coupling delay. (a) The histogram of the ISIs. (b) The membrane potential of two arbitrary neurons *i* = 40 and *j* = 120.

Whether the connections among neurons are partial delayed or all delayed, the above discussion has stated that multiple spatiotemporal orders can appear when the coupling delays are appropriately tuned to about *τ* = 0,720,1440,2160. Moreover, this phenomenon is robust to variations of the fraction of delayed connection and the key parameters of network architecture such as the rewiring probability, the average degree of small-world subnetwork, the initial nodes of scale-free subnetwork and the total size of the modular network. The underlying mechanism of multiple spatiotemporal orders is further explored as follows. When the coupling delay is absent in the modular neuronal network, i.e.,*τ* = 0, the histogram of the ISIs together with the membrane potential of two arbitrary neurons (e.g., *i* = 40,*j* = 120) is depicted in Fig 10. One can observe that the two arbitrary neurons are almost synchronized and their spikes are nearly periodic one. The periodicity is further confirmed to be about 720 by the histogram of ISIs that a sharp peak standing at around 720, which implies that the intrinsic spiking period of the considered modular neuronal network is about 720. Thus, one can conclude that the phenomenon of multiple spatiotemporal orders could be the result of a locking between the length of coupling delay and the intrinsic spiking period of the modular neuronal network.

## Conclusion

Since information communication among neurons in real neural systems can be instantaneous or with some negligible delay. The present study, through investigating the spatiotemporal spiking dynamics of temporal coherence and spatial synchronization, has proposed to explore how the partial coupling delay affects spatiotemporal order in a modular neuronal network. Numerical results have showed the spatiotemporal order that the modular network is most coherent in time and nearly synchronized in space can emerge intermittently when partial coupling delays are appropriately tuned. That is to say, multiple spatiotemporal orders can be induced by partial coupling delay. The phenomenon of multiple spatiotemporal orders is further confirmed to be robust to changes of the fraction of delayed connection, the key parameters of network architecture such as the rewiring probability and the average degree of the small-world subnetwork, the initial nodes of scale-free subnetwork and the total size of the modular network. By analyzing the histogram of the ISIs together with the membrane potential of two arbitrary neurons in the considered modular network, the underlying mechanism of multiple spatiotemporal orders is detected to be the result of a locking between the length of coupling delay and the intrinsic spiking period. Note that the interplay of coupling delay and noise is ubiquitous in neural systems. In addition, the spatial synchronization and temporal coherence of neuronal spike trains are very significant for coding and transmitting information across neural systems. Thus, our results may have important implications, in particular, in understanding information processing in the brain.

## Supporting information

S1 Dataset Data for spatiotemporal plots when only part of connections are delyed. (ZIP)

S2 Dataset Data for quantify spatiotemporal order when only part of connections are delyed. (ZIP)

S3 Dataset Data for variation of the fraction of delayed connection. (ZIP)

S4 Dataset Data for variations of the fraction of delyed connection and the rewiring probability. (ZIP)

S5 Dataset Data for variation of the initial nodes of scale-free subnetwork. (ZIP)

S6 Dataset Data for variation of total size of the modular network. (ZIP)

S7 Dataset Data for spatiotemporal plots when all connections are delyed. (ZIP)

S8 Dataset Data for quantify spatiotemporal order when all connections are delyed. (ZIP)

S9 Dataset Data for variation of the average degree of small-world network. (ZIP)

S10 Dataset Data for the firing dynamics in the absence of coupling delay. (ZIP)
